# A Tet-on and Cre-*lox*P Based Genetic Engineering System for Convenient Recycling of Selection Markers in *Penicillium oxalicum*

**DOI:** 10.3389/fmicb.2016.00485

**Published:** 2016-04-12

**Authors:** Baojie Jiang, Ruiqin Zhang, Dan Feng, Fangzhong Wang, Kuimei Liu, Yi Jiang, Kangle Niu, Quanquan Yuan, Mingyu Wang, Hailong Wang, Youming Zhang, Xu Fang

**Affiliations:** ^1^State Key Laboratory of Microbial Technology, School of Life Science, Shandong UniversityJinan, China; ^2^State Key Laboratory of Microbial Technology, School of Life Science, Shandong University-Helmholtz Institute of Biotechnology, Shandong UniversityJinan, China

**Keywords:** selective markers, *Penicillium oxalicum*, Cre-*lox*P system, Tet-on system, doxycycline

## Abstract

The lack of selective markers has been a key problem preventing multistep genetic engineering in filamentous fungi, particularly for industrial species such as the lignocellulose degrading *Penicillium oxalicum* JUA10-1(formerly named as *Penicillium decumbens*). To resolve this problem, we constructed a genetic manipulation system taking advantage of two established genetic systems: the Cre-*lox*P system and Tet-on system in *P. oxalicum* JUA10-1. This system is efficient and convenient. The expression of Cre recombinase was activated by doxycycline since it was controlled by Tet-on system. Using this system, two genes, *ligD* and *bglI*, were sequentially disrupted by *lox*P flanked *ptrA*. The successful application of this procedure will provide a useful tool for genetic engineering in filamentous fungi. This system will also play an important role in improving the productivity of interesting products and minimizing by-product when fermented by filamentous fungi.

## Introduction

Filamentous fungi express and secrete valuable primary and secondary metabolites, playing an important role in biotechnology (Gerngross, 2004; Nevalainen and Peterson, 2014; Chávez et al., 2015). *Penicillium* species are among the best known important strain in industry. *Penicillium* species have been used widely for producing various products since 1930s, including penicillins (Nijland et al., 2008; Houbraken et al., 2012; Weber et al., 2012) and various cellulolytic enzymes (Adsul et al., 2007; Jung et al., 2015; Liao et al., 2015; Sharma et al., 2015). More than 250 *Penicillium* species have been identified currently (Houbraken and Samson, 2011) and lots of interesting metabolites are being explored. *P. oxalicum* was identified with strong cellulolytic ability and has been investigated for more than 30 years in China. Therefore, an efficient and convenient genetic engineering system should be developed in *P. oxalicum*. Many genetic engineering methods for gene disruption or insertion rely heavily on selection markers for screening target transformants. In filamentous fungi, several selection marker genes (SMGs) such as hygromycin B resistance gene (*hph*) (Michielse et al., 2005), pyrithiamine resistance gene (*ptrA*) (Wang F. Z. et al., 2013), and phleomycin resistance gene (Lecordier et al., 2015) are in use currently. However, the markers cannot satisfy the need for complex genetic engineering in one strain. The limitation has hampered the progress of genetic engineering in filamentous fungi.

This limitation has been resolved by taking advantage of the Cre-*lox*P system used to recycle SMGs in other species. Cre recombinase (Cre) is a member of the bacteriophage λ-integrase family of recombinases which covalently binds to two 34 bp *lox*P sites and catalyzes recombination between them (Pinkney et al., 2012; Gaj et al., 2014). Cre-*lox*P system has been used extensively to study gene function and to remove selection markers in many organisms, including mammals such as mouse (Tang et al., 2002; Chen et al., 2009), plants such as tobacco (Luo et al., 2007) and *Arabidopsis thaliana* (De Buck et al., 2007). Cre-*lox*P system is also successfully used in fungi such as *Trichoderma reesei* (Steiger et al., 2011), *Saccharomyces cerevisiae* (Yamagishi et al., 2008), and *Aspergillus* species (Forment et al., 2006). In the Cre-*lox*P system, the constitutive expression of Cre will affect the recycling of SMGs flanked by *lox*P. Therefore, controlled expression of Cre is necessary. In *Saccharomyces* spp., genes can replicate and express autonomously in episomal plasmid without integration into genome, and the plasmid is deleted when grown on non-selective medium (Aoki et al., 2010). However, there is no episomal plasmid reported for filamentous fungi. Steiger et al. used *xylanase 1* promoter to control expression of Cre in *T. reesei* (Steiger et al., 2011). However, Ogasawara et al. (2006) reported that xylanase promoters are induced by many kinds of carbohydrates, including _*D*_-xylose, Avicel, _*L*_-sorbose, sophorose, xylobiose, xylan, cellulose, and sophorose. Derntl et al. reported that the change of xylanase expression has a significant influence on cellulase production (Derntl et al., 2013). Thus, the method with controlling expression of Cre using xylanase promoters is not suitable for Cre-*lox*P system in cellulase-producing fungi.

The tetracycline (Tet)-inducible system has been used widely to control the expression of transgene efficiently (Barde et al., 2006; Delerue et al., 2014). In the Tet-on system, the reverse tetracycline transactivator (rtTA) interacts with Tet resistance operon (*tetO*) in the presence of tetracycline or its derivative doxycyline and activates transcription (Stieger et al., 2009). Tet-on system has been used in many species, including mice (Furth et al., 1994; Delerue et al., 2014); *Plasmodium falciparum* (Dahl et al., 2006) and fungal species such as *Candida albicans* (Park and Morschhäuser, 2005), *Schizosaccharomyces pombe* (Faryar and Gatz, 1992), and *A. fumigatus* (Vogt et al., 2005). Doxycycline has been used to regulate gene expression successfully in *C. albicans* (Nakayama et al., 2000) and *A. niger* (Meyer et al., 2011) and has been reported to be the most effective in regulation of tetracycline-regulated promoters in *S. Cerevisiae* (Garí et al., 1997). *P. oxalicum* JUA10-1 has been used for the production of lignocellulytic enzymes with good performance at industrial-scale (Wang M. Y. et al., 2013). However, limited genetic modification work was carried out due to the non-recycle use of selection markers.

In this study, we describe a modified Cre-*lox*P system under the control of Tet-on system for marker gene excision in *P. oxalicum* JUA10-1. In this system, the expression of Cre is transient only after induction by doxycycline. Since *ptrA* can be reused, multiple genetic engineering could be carried out to modify *P. oxalicum*. This system will provide a platform for genetic engineering in filamentous fungi.

## Materials and methods

### Strains and cultivation conditions

*P. oxalicum* JUA10-1, which is the catabolite-repression-resistant mutant of *P. oxalicum* strain 114-2 (CGMCC 5302), is a cellulase hyper-producing strain maintained in our laboratory (Wang M. Y. et al., 2013). All *P. oxalicum* strains used in this study are listed in Table 1. The modified Mandels' salt solution (MMs) was used as previous described (Liu et al., 2013). The MMs used for cultivation *P. oxalicum* contained 2% (wt/vol) glucose (GMM), and uridine (10 mM) was added when required. For cellulase protein production of *P. oxalicum*, the strains were cultivated in liquid MMs added with Avicel (6 g/l), wheat bran (30 g/l), peptone (5 g/l), and tween-80 (3 ml/l).

**Table 1:** ***P. oxalicum* strains used in this study**.

**Strain**	**Genotype**	**Source**
JUA10-1	Parent strain	This laboratory
JUA-*ΔpyrG*	Derived from JUA10-1; deletion of *pyrG* using *hph*; containing Cre controlled by Tet-on system	This work
JUA-*ΔligD*	Derived from JUA-*ΔpyrG*; deletion of *ligD* using *lox*P flanked *ptrA*	This work
JUA-*ΔligDΔptrA*	Derived from JUA-*ΔligD*; excision of *ptrA* by Cre	This work
JUA-*ΔbglI*	Derived from JUA-*ΔligDΔptrA*; deletion of *bglI* using *lox*P flanked *ptrA*	This work

*E.coli* strain GB05-dir (Fu et al., 2012) was maintained in Luria-Bertni medium containing appropriate antibiotic (chloramphenicol 15 μg/ml, 100 μg/ml ampicillin) at 37°C.

### Plasmids construction

All primers used in this study are listed in Table 2. PCR amplification was performed using KOD FX DNA polymerase (Toyobo Co., Ltd., Osaka, Japan). The conditions for PCR amplification followed the protocols described by the manufactures.

**Table 2:** **Primers used in this study**.

**Primer**	**Sequence (5′-3′)**
G1	CGATTTAGGTGACACTATAGAACGCGAGGAATTTGAAACTGCGGCTGCAC
G2	ACATCTGCTGTCAAAGCTGGAGCTCCACCGCGGTGG
G3	AGCTCCAGCTTTGACAGCAGATGTGATCGGAGACAA
G4	GGACCCAGCTGTGGACTATCGTAGTAGAACCCAGGGGCTGGTGACGGAAT
G5	ATTCCGTCACCAGCCCCTGGGTTCTACTACGATAGTCCACAGCTGGGTCC
G6	TAGACATGGTGAGGTTAAGAGGGTTCTTCCGGCTTCG
G7	ACCCTCTTAACCTCACCATGTCTAGACTGGACAAGAGC
G8	GGCTCTGGTTGAGGTGATGTCTGCTCAAGCGGGGTA
G9	ACACGTGAATTCGGCCGCGGATCTGCCGGTCTCCCT
G10	TCCACGAACCTTTGAATTCAGACTCCTGAAGATCTTCCGTTAATGGCTAA
G11	TTAGCCATTAACGGAAGATCTTCAGGAGTCTGAATTCAAAGGTTCGTGGA
G12	GCAGACATCACCTCAACCAGAGCCGATCCTGTACACG
G13	AGATCCGCGGCCGAATTCACGTGTGGTAGGACTA
G14	GTGCAGCCGCAGTTTCAAATTCCTCGCGTTCTATAGTGTCACCTAAATCG
ptrA-F	GAAGATCTGGGCAATTGATTACGGGATCCCATTGG
ptrA-R	CCCTCGAGATGGGGTGACGATGAGCCG
L1	CGATTTAGGTGACACTATAGAACGCCTCGATATGCTTCAGGCCATGCTCC
L2	CGTTTCCGTCATGTAGCAATCACAGAGCTTCGTACGCTGCAGGTCGACAAC
L3	GTTGTCGACCTGCAGCGTACGAAGCTCTGTGATTGCTACATGACGGAAACG
L4	GTTGAGATATTCCCCGTCTGTGCGTGCATAGGCCACTAGTGGATCTGATA
L7	TATCAGATCCACTAGTGGCCTATGCACGCACAGACGGGGAATATCTCAAC
L6	CGAATGCGCTCGTCATGGGAGAATCGGCCGCGGATCTGCCGGTCTCCCTAG
L5	TAGGGAGACCGGCAGATCCGCGGCCGATTCTCCCATGACGAGCGCATTC
L8	CGATTTAGGTGACACTATAGAACGCCTCGATATGCTTCAGGCCATGCTCC
B1	ACTGGGTATTTCGGGTAGCTTCCACTCAGTGTAGGAGGATGCGGGGAGAG
B2	CTCTGTGATTGCTACATGACGGAAGGTTCTTGCCCAATGGACCAGCGACG
B3	CGTCGCTGGTCCATTGGGCAAGAACCTTCCGTCATGTAGCAATCACAGAG
B4	GTATCACACTCGACCTGACAGTTGCGTTCATCATTTACTGCACCTTGGGC
B5	GATGGGAAAGCTGTGGGATACTAGGTGCCCGGTGATGTGATTCTTGGTAG
B6	CTACCAAGAATCACATCACCGGGCACCTAGTATCCCACAGCTTTCCCATC
B7	GCCCAAGGTGCAGTAAATGATGAACGCAACTGTCAGGTCGAGTGTGATAC
B8	CTCTCCCCGCATCCTCCTACACTGAGTGGAAGCTACCCGAAATACCCAGT
B9	CATCCGCATCATCCGCATAGCCCAAAGC
B10	GTCCGGACGAGCCTTGGCCAAGACGT
GS1	CGACCGAACTTGTACTGGCAGATTG
GS2	CTTTCAGCCTCCATTGTAGCCTCCA
LS1	GAAGCATGGAAGTTATCGTGGGACCAGTC
LS2	ACTGCCATTGGATAGCCTGTTGTCGATC

**Figure 1:**
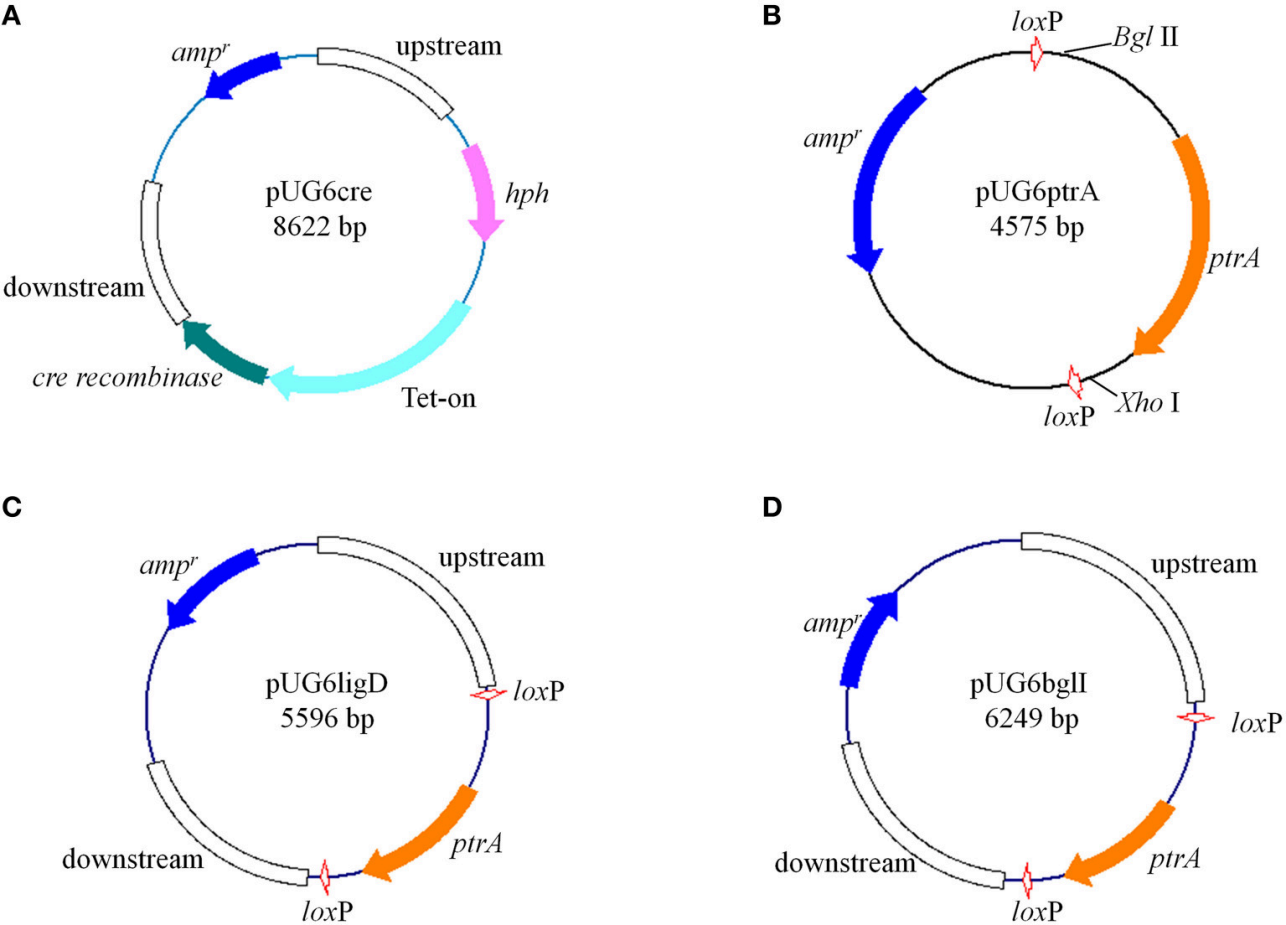
**Structures of plasmids constructed in this study (described in “Materials and Methods”) (A) pUG6cre; (B) pUG6ptrA; (C) pUG6ligD; (D) pUG6bglI**.

The Cre expression plasmid pUG6cre (Figure 1A) was constructed by linear-linear homologous recombination mediated by full length RecET in GB05-dir (Fu et al., 2012; Yin et al., 2015). The fragments were obtained as follows. The up- and down-stream of the orotidine 5′-phosphate decarboxylase gene (*pyrG*, GenBank: GU574814.1) were PCR amplified from *P. oxalicum* genome using primer pairs G1/G2 and G11/G12, respectively. Hygromycin B resistance gene (*hph*) with its promoter and terminator was obtained using primer pairs G3/G4 and plasmid pSilent-1 (Nakayashiki, 2005) as template. *T. reesei* pyruvate kinase gene promoter (P*pki1*), which was used to replace P*gpdA* in Tet-on system, was obtained using primer pairs G5/G6 from *T. reesei* genome. Tet-on system without P*gpdA* was amplified by PCR from plasmid pVG2.2 (Ouedraogo et al., 2011) with primer pairs G7/G8, and Cre-encoding sequence was obtained from plasmid pSH65 by PCR with primer pairs G9/G10. The ampicillin resistance gene and replication initiation site was amplified by PCR from pUG6 using primer pairs G13/G14.

For construction of the *lox*P*-ptrA*-*lox*P cassette, the *ptrA* gene was inserted into plasmid pUG6 between two *lox*P sites (Figure 1B). Firstly, the *ptrA* fragment containing its promoter and terminator was amplified using primers BglIIptrAfw (containing *Bgl* II recognition sequence) and ptrArevXhoI (containing *Xho* I recognition sequence). The plasmid pUG6 was then digested with *Bgl* II-*Xho* I and the *ptrA* fragment was ligated into plasmid pUG6 using the corresponding restriction sites, yielding plasmid pUG6ptrA.

The gene encoding *P. oxalicum* Ligase IV homolog, Ligase D (*ligD*, GenBank: EPS32530.1) deletion plasmid pUG6ligD (Figure 1C) was constructed based on plasmid pUG6ptrA. The up- and down-stream regions of the *ligD* were obtained by PCR from *P. oxalicum* genome with primer pairs L1/L2 and L5/L6, respectively. The *ptrA* fragment containing two *lox*P sites were amplified by PCR from plasmid pUG6ptrA with primer pairs L3/L4. The vector fragment containing ampicillin resistance gene and replication initiation site was PCR amplified from pUG6ptrA using primer pairs L7/L8. After purification, the four fragments were used to construct plasmid pUG6ligD in GB05-dir by linear-linear homologous recombination.

The construction of β-glucosidase gene (*bglI*, GenBank: EU700488.1) deletion plasmid named pUG6bglI (Figure 1D) was similar to the construction of plasmid pUG6ligD. The up- and down-stream regions of the *bglI* were obtained by PCR from *P. oxalicum* genome with primer pairs B1/B2 and B5/B6, respectively. The *ptrA* fragment and the vector fragment were obtained from pUG6ptrA using primer pairs B3/B4 and B7/B8, respectively. The plasmid pUG6bglI was then constructed by linear-linear homologous recombination in GB05-dir.

### Transformation of *P. oxalicum* and analysis

**Figure 2:**
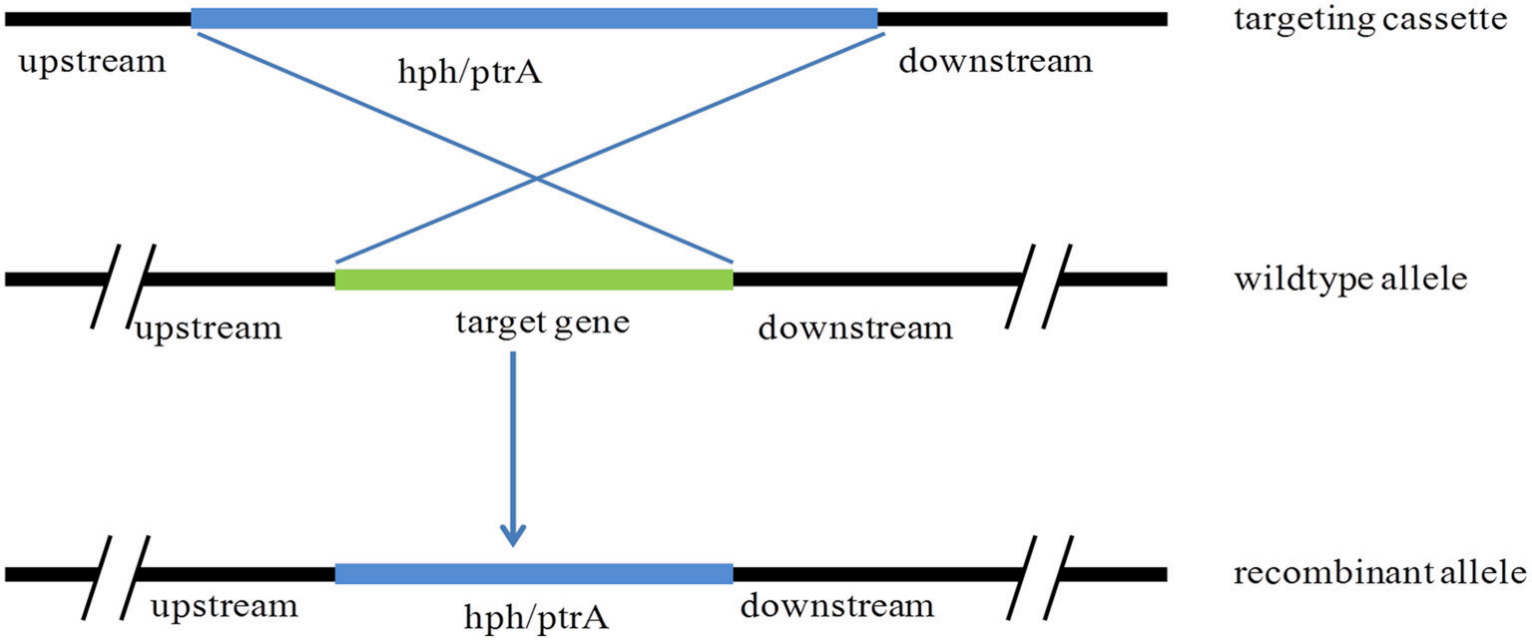
**Influence of doxycycline concentrations on colony diameter of *P. oxalicum* spotted on GMM plate at 30°C**. Concentrations of doxycycline (μg/ml): open squar, 0; open triangle, 30; open circle, 50; closed squar, 100; closed triangle, 200; closed circle, 300.

The transformation of *P. oxalicum* was carried out using the method as previous described (Li et al., 2010). The plasmids, pUG6cre, pUG6ligD, and pUG6bglI, were transformed into *P. oxalicum* JUA10-1 sequentially. The Cre recombinase under control of Tet-on system and the *lox*P flanked *ptrA* were introduced into the *P. oxalicum* chromosome through homologous recombination method (Figure 2).The plasmid pUG6cre was used to introduce Cre recombinase under control of Tet-on system in site of *pyrG* through homologous recombination. The plasmid pUG6ligD was used to introduce *lox*P flanked *ptrA* in site of *ligD* through homologous recombination. And the plasmid pUG6bglI was used to introduce *lox*P flanked *ptrA* in site of *bglI* through homologous recombination. After cultivation at 30°C for 5–7 days, the candidate transformants were selected on solid GMM with 10 mM uridine containing 350 μg/ml hygromycin B or 0.3 μg/ml pyrithiamine.

The genomes of candidate transformants were extracted and screened by PCR. To further confirm the validity of the transformants, Southern blotting analysis was performed according to the manufacturer's instruction of a DIG Easy Hyb kit (Roche Diagnostics, Germany). Probe used to analyze deletion of *pyrG* with primer pairs GS1/GS2 was amplified from *P. oxalicum* genome. Probe used to analyze disruption of *ligD* and excision of *ptrA* was amplified from *P. oxalicum* genome with primer pairs of LS1/LS2.

Transformants were selected by growth on GMM plate containing 350 μg/ml hygromycin B or 0.3 μg/ml pyrithiamine.

### Expression of Cre and excision of *ptrA*

Tet-on system induced by the doxycycline was ligated with Cre-encoding sequence for controlled expression of Cre. For expression of Cre, about 100 conidia of the positive transformant containing Cre expression cassette and *lox*P-*ptrA*-*lox*P cassette were inoculated on GMM plate added 50 μg/ml doxycycline (Sigma-Aldrich, St. Louis, MO, USA) for 1 week. The colonies were screened by PCR to test the excision of *ptrA*.

### β-glucosidase activity assay and protein quantification

β-glucosidase activity was measured using 5 mM _*p*_-nitrophenyl-β- _*D*_-glucopyranoside (*p*NPG) (Sigma-Aldrich, St. Louis, MO, USA) in 5 mM sodium citrate buffer, pH 4.8 as substrate. Fifty microliter substrate and 100 μl sample were incubated at 50°C for 30 min, 150 μl of 10% (wt/vol) Na_2_CO_3_ was added to terminate the reaction. Absorbance was read at 420 nm in a microplate using a plate reader (Infinite 200 NanoQuant, Tecan, Zurich, Switzerland)._**p**_-nitrophenol was used to prepare a standard curve. One unit (IU) of enzyme activity was defined as the amount of enzyme needed to hydrolyze 1 μmol *p*NPG in 1 min.

Protein quantification was measured using Bradford with bovine serum albumin (BSA) as standard (Bradford kit, Sangon Biotech, Shanghai, China).

## Results

### Effects of doxycycline on the growth of *P. oxalicum*

**Figure 3:**
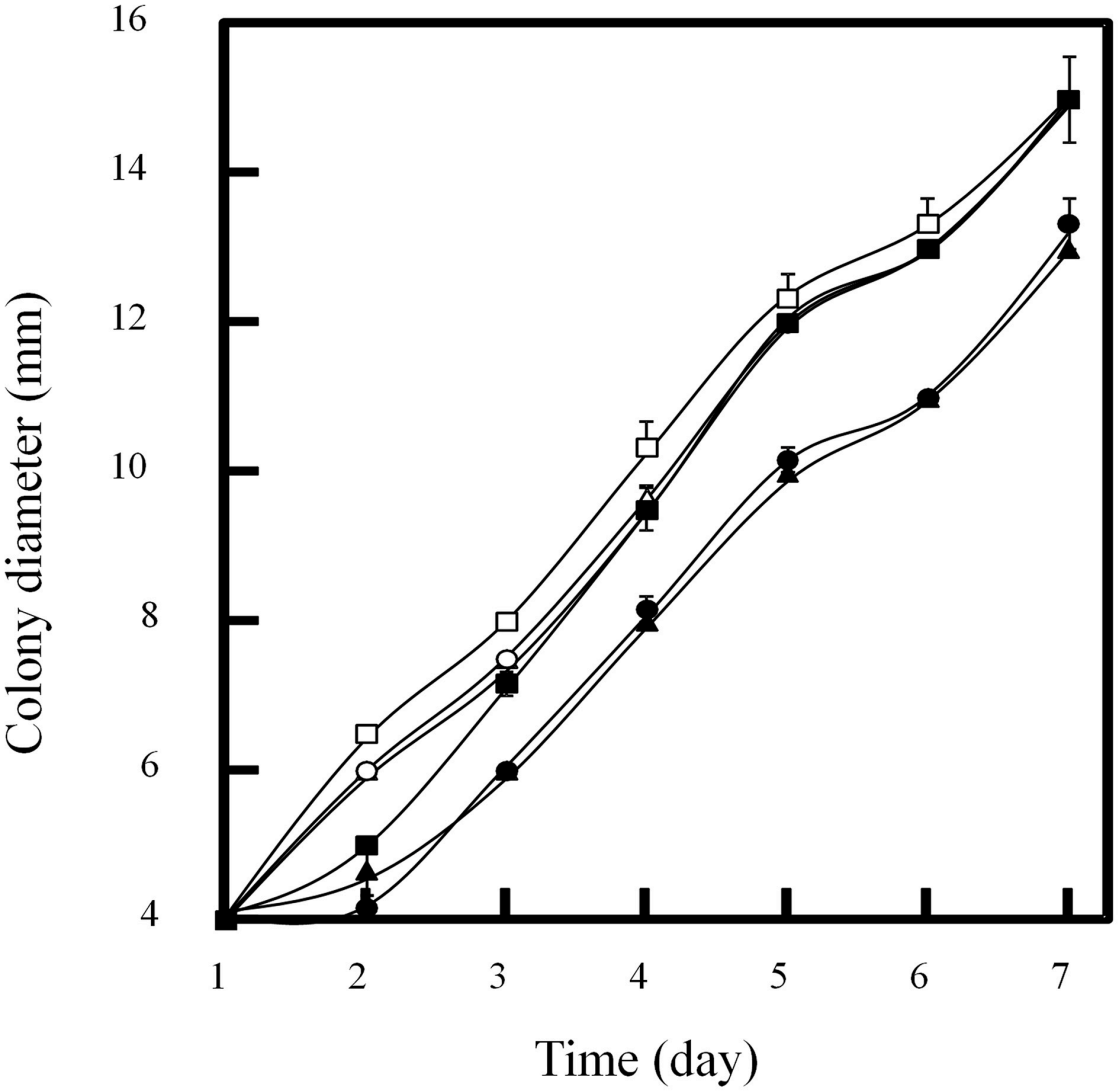
**Schematic of homologous recombination in *P. oxalicum***.

In order to determine the tolerance of *P. oxalicum* to doxycycline, about 10^3^ conidia were inoculated on GMM plate containing 0–300 μg/ml of doxycycline. The plates were incubated at 30°C for 7 days and the diameters of colonies were measured. There was no significant influence on growth of *P. oxalicum* when 100 μg/ml of doxycycline was used (Figure 3). However, when the concentration of doxycycline increased to 200 μg/ml, the diameters of colonies decreased from 15 to 13 mm at the 7th day. Therefore, there is no significant influence on *P. oxalicum* growth when doxycycline under concentration of 100 μg/ml was added.

### Introduction of Cre under control of tet-on system in *P. oxalicum*

The Cre gene expression cassette for deletion *pyrG* was induced into JUA10-1 with plasmid pUG6cre. The *pyrG* deletion strain is uridine auxotrophy, thus candidate transformants were grown on GMM plate containing 10 mM uridine. After selection on GMM plate containing 350 μg/ml hygromycin B, the candidate transformants were screened by PCR to detect Cre-encoding sequence with primer pairs G9/G10. We chose one correct strain, called JUA-Δ*pyrG*, and confirmed the deletion of *pyrG* in the JUA-Δ*pyrG* strain using Southern blotting analysis (Figure S1A). The genomes of parent and JUA-Δ*pyrG* strains were digested by restriction enzyme of *Kpn* I. Southern blotting analysis showed that the parent chromosome generated a 7.5 kb fragment, while the chromosome of *pyrG* deletion strain generated a 3.7 kb fragment. The result confirmed that chromosome of JUA-Δ*pyrG* contains one copy of Cre expression cassette and *pyrG* was deleted completely.

### Deletion of *ligD* using *lox*P flanked *ptrA* in *P. oxalicum*

Furthermore, the *ligD* gene of JUA-Δ*pyrG* strain was deleted by *lox*P flanked *ptrA* through homologous recombination using plasmid pUG6ligD to improve the homologous recombination frequency with destruction of NHEJ pathway. After selected on GMM plates containing 0.3 μg/ml pyrithiamine and 10 mM uridine, candidate transformants were analyzed by PCR to amplify *ptrA* fragment with primer pairs L3/L4. We chose one of the correct strains, called JUA-Δ*ligD*, and then confirmed the deletion of *ligD* by Southern blotting analysis. The genomes of the JUA-Δ*pyrG* strain and the JUA-Δ*ligD* strain were extracted for Southern blotting analysis. The result of Southern blotting was shown in Figure S1B. After digestion by restriction enzyme of *Bgl* II, the JUA-Δ*pyrG* strain generated a 7.1 kb fragment, while the JUA-Δ*ligD* strain generated a 4.6 kb fragment. Our result showed that the JUA-Δ*ligD* strain contained one copy of deletion cassette accompanied by the complete deletion of the *ligD* allele.

### Expression of Cre and excision of *ptrA*

**Figure 4:**
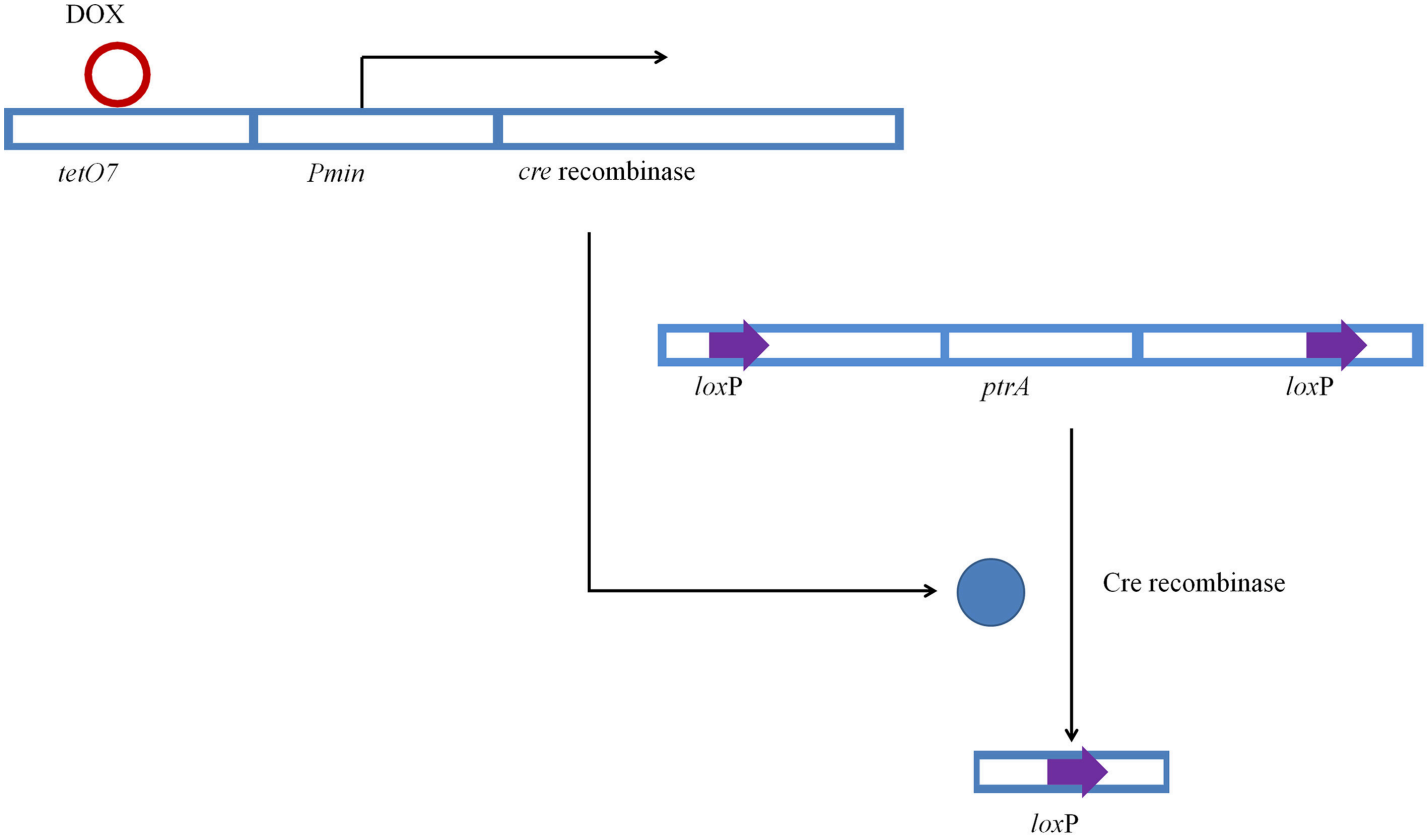
**Principle of the Cre-*lox*P system under the control of Tet-on system in *P. oxalicum***.

Conidia of JUA-Δ*ligD* strain was inoculated on GMM plates containing 50 μg/ml of doxycycline and 10 mM uridine to induce expression of Cre, after which the expressed Cre excised *ptrA*. We also inoculated JUA-Δ*ligD* strain on GMM plate without doxycycline to test whether the expression of Cre was accurately controlled by Tet-on system. Figure 4 showed the schematic diagram of introduction of Cre and excision of *ptrA* in JUA-Δ*ligD* strain.

After induction of doxycycline, 20 strains were chosen to extract genomes. PCR with primer pairs L3/L4 was carried out to test the excision of *ptrA*. The result showed that four of these strains exicised the *ptrA*. The frequency of excision was about 20%. We chose one of the JUA-Δ*ligD* strains with *ptrA* excised, and called it JUA-Δ*ligD*Δ*ptrA*. Southern blotting analysis was then carried out to further verify deletion of *ptrA*, as shown in Figure S1C. After digestion of genetic DNA with restriction enzyme of *EcoR* I, the strain without induction by doxycycline generated an 8.8 kb fragment, while the doxycycline-induced strain generated a 2.8 kb fragment. The result showed that *ptrA* was completely excised by Cre under the operation of Tet-on system in the presence of doxycycline induction only.

**Figure 5:**
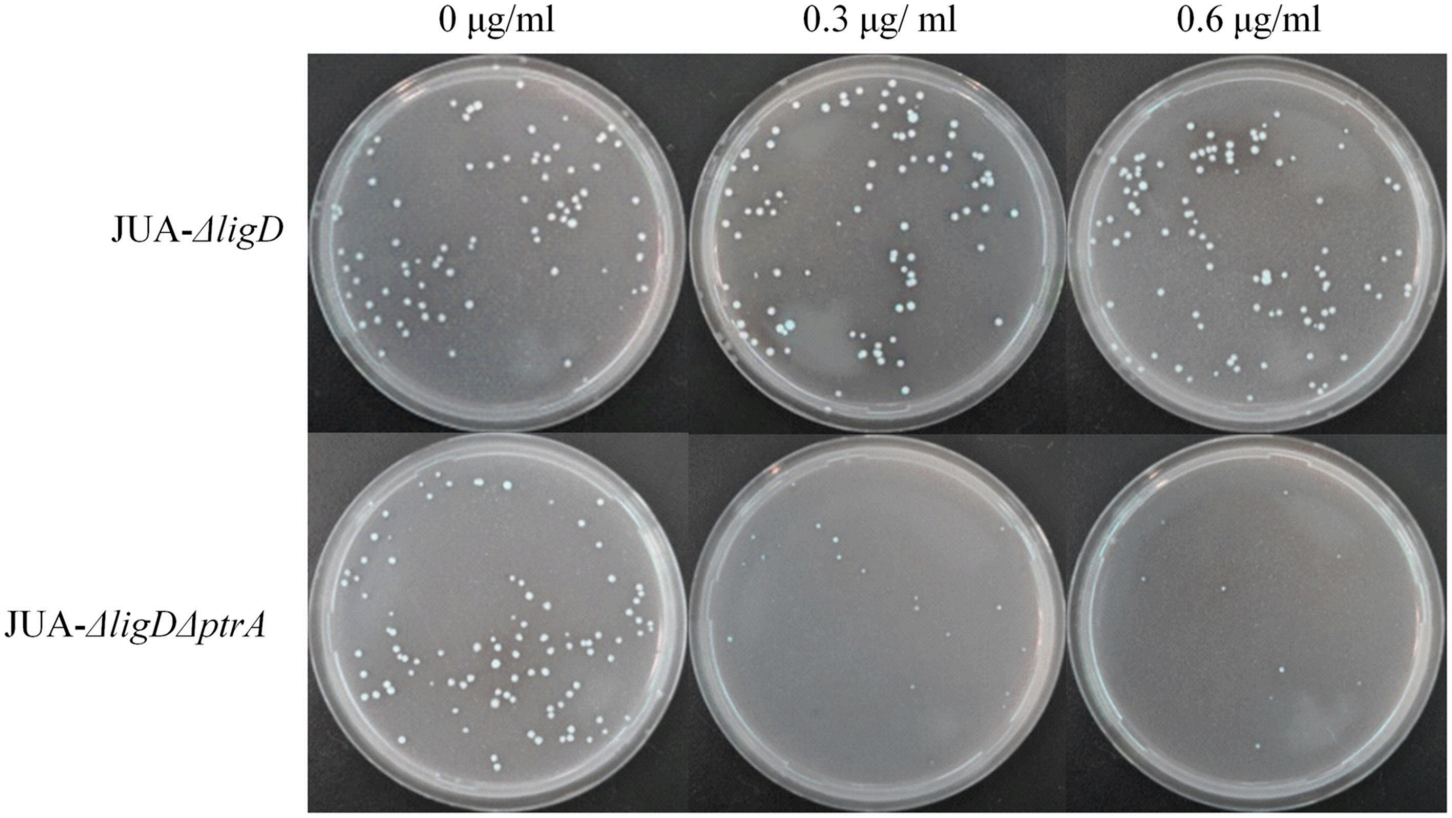
**Resistance to pyrithiamine of JUA-Δ*ligD* and JUA-Δ*ligD*Δ*ptrA* strains**. Concentration of pyrithiamine: 0, 0.3, 0.6 μg/ml.

About 100 conidia of JUA-Δ*ligD* and JUA-Δ*ligD*Δ*ptrA* strains were inoculated on GMM plates containing 0, 0.3, or 0.6 μg/ml of pyrithiamine and 10 mM uridine. We found that the JUA-Δ*ligD* strains containing *ptrA* had a normal growth after 5 days cultivation. Meanwhile, the colony numbers of JUA-Δ*ligD*Δ*ptrA* on the plates decreased as the concentration of pyrithiamine increased (Figure 5). The result further suggested that the *ptrA* was excised completely by Cre after induction of doxycycline.

These results suggested that the expression of Cre is controlled by Tet-on system efficiently.

### Reuse of *ptrA* and deletion of *bglI*

**Figure 6:**
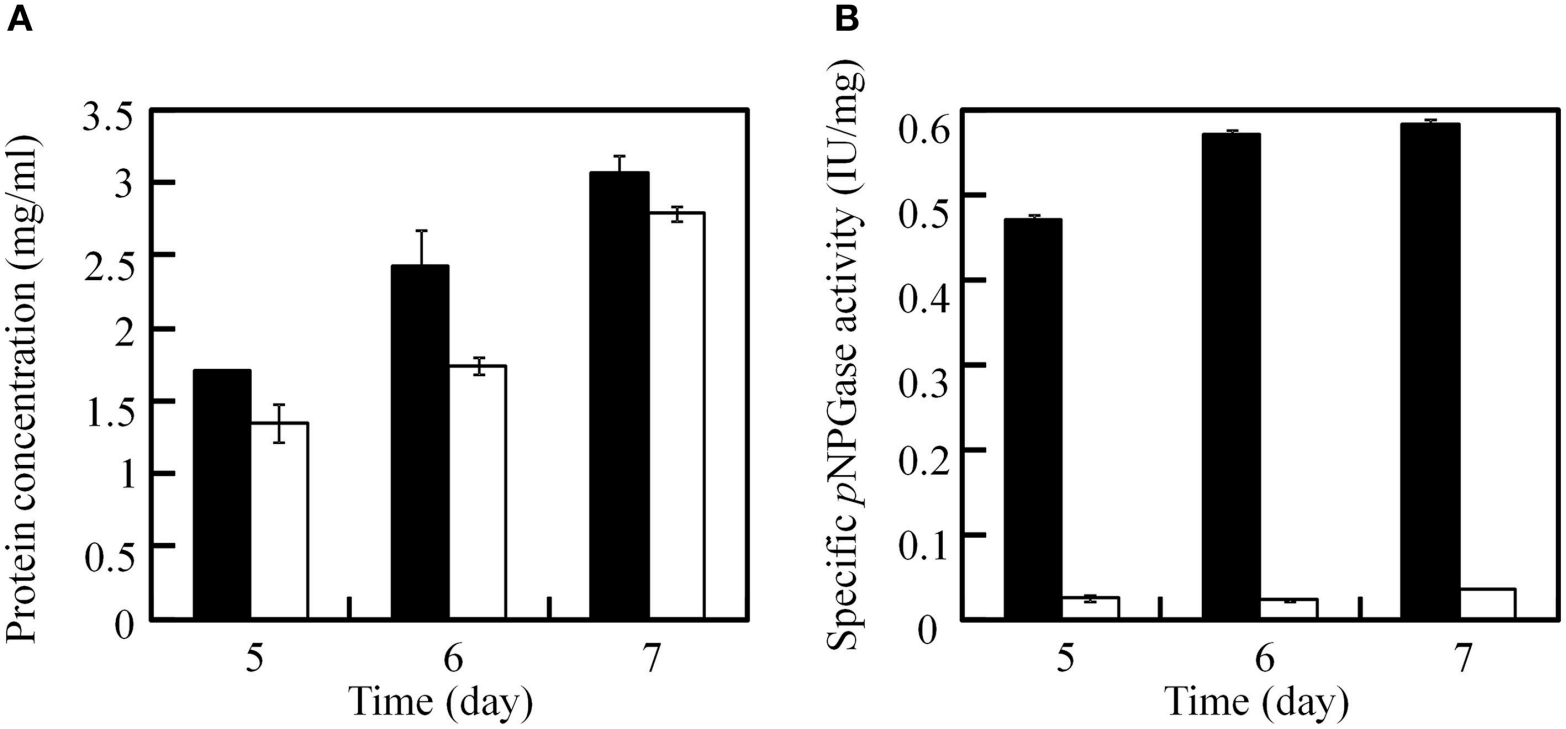
**Comparison of soluble protein concentration (A) and specific β-glucosidase activity (B) between JUA-Δ*ligD*Δ*ptrA* and JUA-Δ*bglI* strains**. The solid bar represents JUA-Δ*ligD*Δ*ptrA* strain, the hollow bar represents JUA-Δ*bglI* strain.

After excision of the *ptrA*, the β-glucosidase (*bglI*) gene in the JUA-Δ*ligD*Δ*ptrA* strain was disrupted by reusing *ptrA* to testify the recycle use of *ptrA* because it was easy for measuring the β-glucosidase activity. After transformation with plasmid pUG6bglI, 13 candidate transformants were selected on GMM plates containing 0.3 μg/ml pyrithiamine and 10 mM uridine. PCR analysis was used to test the insertion of *ptrA* fragment with primer pairs B3/B4 and the result proved that five transformants contained *ptrA*. Furthermore, another primer pairs B9 (upstream of *bglI* deletion cassette) and B10 (inside of *ptrA*) were used to testify the homologous recombination of *ptrA* using PCR method. The results showed that *ptrA* was inserted into *bglI* locus in all five transformants. One correct strain was chosen and called JUA-Δ*bglI*. Then, we measured the soluble protein concentration and *p*NPGase activities in JUA-Δ*ligD*Δ*ptrA* and JUA-Δ*bglI* strains at 5, 6, and 7 days. As shown in Figure 6, there was no significant change of soluble protein concentrations between the two strains, while the specific *p*NPGase activity in JUA-Δ*bglI* strain was greatly lower than that of JUA-Δ*ligD*Δ*ptrA* strain. After cultivation for 7 days, the specific *p*NPGase activate of JUA-Δ*bglI* strain was merely 0.034 IU/mg of soluble protein, only 5.8% of that of JUA-Δ*ligD*Δ*ptrA* strain (0.58 IU/mg of soluble protein). The result suggested that β-glucosidase encoding gene was disrupted by *ptrA* successfully.

## Discussion

With the broad usage of filamentous fungi, genetic engineering is urgent to modify filamentous fungi, especially for industrial strains. Although many technologies have been employed for genetic engineering for filamentous fungi since the discovery of DNA double helix, only one or two of these technologies are available for a particular species. Recently, the CRSPR-Cas9 system has been used for genetic editing with high efficiency in filamentous fungi. Liu et al. (2015) reported that CRISPR/Cas9 system has been applied for genetic editing in *T. reesei* and then the promoter of *cbh1*(Pcbh1) was used to control the expression of Cas9. However, the expression of Cas9 with Pcbh1 has a negative influence on cellulase synthesis. Therefore, it is not suitable for cellulase-producing strains, such as *T. reesei* and *P. oxalicum*. Meanwhile, the Tet-on system has no negative effect on cellulase synthesis. Furthermore, the CRSPR/Cas9 system still relies on SMGs for multiple genetic operations, and on the contrary, the Tet-on and Cre-*lox*P system reduces the limitation of SMGs. In all, the Tet-on and Cre-*lox*P system still has an advantage in the homologous recombination.

Construction of a system to recycle SMGs is crucial in the extensive engineering of filamentous fungi. The Cre-*lox*P system has been widely used for filamentous fungi to rescue available SMGs (Mizutani et al., 2012; Zhang et al., 2013; Krappmann, 2014). However, there is no such report of Cre-*lox*P system used in *P. oxalicum*. In this paper, we successfully constructed a Cre-*lox*P system controlled by Tet-on system, and *ptrA* gene was excised successfully in *P. oxalicum* for multiple engineering operations.

The accurate control of the expression of Cre is the key in Cre-*lox*P system to excise the SMGs. During the genetic operation, the constitutive expression of Cre will affect the next gene targeting using *lox*P flanked marker gene. Therefore, an inducible promoter controlling the expression of Cre should be used. Steiger et al. used *xylanase 1* promoter to control expression of Cre in *T. reesei* (2011). However, the xylanase promoters can be induced by degradation products of lignocelluloses (Ogasawara et al., 2006). Therefore, xylanase promoters are not suitable for cellulase-producing filamentous fungi. Florea et al. used a Cre-expressing plasmid to transiently transfect the target strain (Florea et al., 2009). However, the efficiency was so low that only 0.5–2% of colonies eliminated marker gene with this method. Meanwhile, additional work was needed to transfect the target strain for expression of Cre every time. Zhang et al. constructed a Cre-*lox*P system to recycle marker gene via anastomosis method in *Cryphonectria parasitica* (Zhang et al., 2013). In that method, the Cre gene was controlled by a constitutive promoter. One strain was used as the donor to produce Cre. The other strain containing *lox*P flanked marker gene was used as recipient strain. The SMGs of recipient strain was deleted by Cre from the donor strain with co-cultivation. However, this method was very complicated and time-consuming.

In this study, we used the Tet-on system as the operator to control expression of Cre. The Tet-on system was turned on by induction of doxycycline, and the expression of Cre is activated. After removing doxycycline, the Tet-on system was turned off. This procedure is efficient and convenient. Firstly, the procedure does not need additional transformation for excision of *lox*P flanked marker gene described as the method by Florea et al. (2009). After cultivation on doxycycline medium, the frequency of excision of *ptrA* is about 20%, more efficient than the method reported by Florea et al. (2009). And the colonies do not need subculture described as the method by Zhang et al. (2013). Thus, it will be time-saving when extensive genetic engineering work is carried out. Secondly, the expression of Cre is efficiently controlled by the Tet-on system induced by doxycycline. The Tet-on system has been widely used in many species, such as mouse (Milo-Landesman et al., 2001; Giménez et al., 2004), zebrafish (Li et al., 2012), and *C. albicans* (Oliver et al., 2008). As the inducer of rtTA, the induction of doxycycline has been shown efficiently (Berenjian and Akusjärvi, 2006; Myoung and Ganem, 2011; Chen et al., 2015). Our result showed that Cre was not expressed without induction of doxycycline as shown in Figure S1C. It suggested that the control of Cre-*lox*P system by Tet-on system was entirely feasible.

In conclusion, we present a Cre-*lox*P plus Tet-on system for genetic engineering with markerless gene deletion in *P. oxalicum.* Markerless gene deletion technology is expected to apply for industry strains. The *ligD* and *bglI* genes were sequentially knocked out by selection marker *ptrA* using this system. The *ptrA* gene was excised by Cre completely and can be reused. Comparing with other Cre-*lox*P systems in filamentous fungi, this system is efficient and convenient. This modified Cre-*lox*P system will provide an excellent platform for genetic engineering in filamentous fungi.

## Author contributions

BJ carried out the experiments, analyzed the data, and wrote the draft. XF designed the experiments, analyzed the data, and wrote the manuscript. RZ, DF, FW, KL, YJ, KN, and QY carried out the experiments. MW, HW, and YZ reviewed the paper. All the co-authors have read the manuscript and agreed to publish.

### Conflict of interest statement

The authors declare that the research was conducted in the absence of any commercial or financial relationships that could be construed as a potential conflict of interest.

## Supplementary material

Figure S1 **Southern blotting analysis of genes disruption in *P. oxalicum*. (A)** Southern blotting analysis of deletion of *pyrG* in *P. oxalicum*. **(B)** Southern blotting analysis of deletion of *ligD*. **(C)** Southern blotting analysis of excision of *ptrA*.
