# Supplementary figures and images for: A Tet-on and Cre-loxP Based Genetic Engineering System for Convenient Recycling of Selection Markers in Penicillium oxalicum
Source: Front Microbiol. 2016 Apr 12;7:485. doi: 10.3389/fmicb.2016.00485 (PMC4828452; doi:10.3389/fmicb.2016.00485)

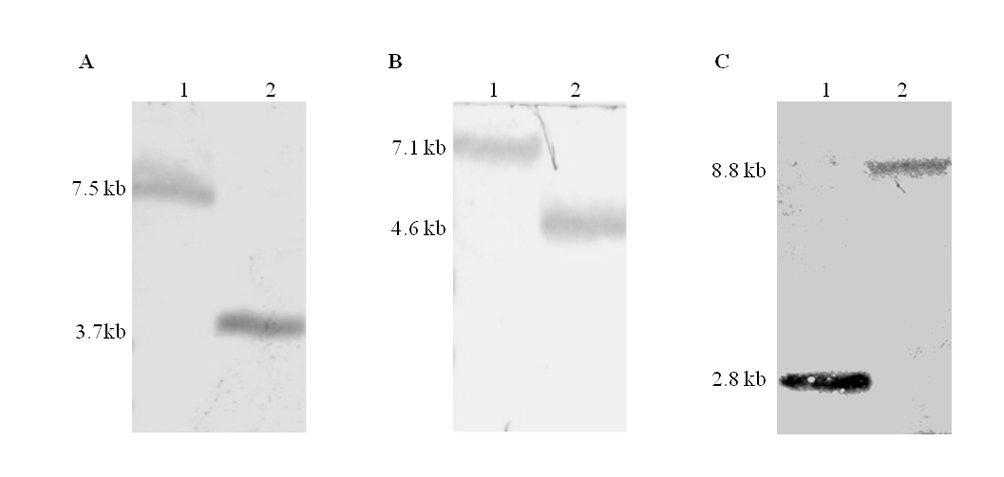

Supplement: Figure S1 — Southern blotting analysis of genes disruption in P. oxalicum. (A) Southern blotting analysis of deletion of pyrG in P. oxalicum. (B) Southern blotting analysis of deletion of ligD. (C) Southern blotting analysis of excision of ptrA. [file FigureS1.TIF]
